# Antonio Scarpa (1752–1832)

**DOI:** 10.1007/s00415-012-6658-4

**Published:** 2012-08-29

**Authors:** Andrzej Grzybowski, Jarosław Sak

**Affiliations:** 1 Department of Ophthalmology, Poznań City Hospital, ul. Szwajcarska 3, 61-285 Poznań, Poland; 2 Department of Ophthalmology, University of Warmia and Mazury, Olsztyn, Poland; 3 Department of Ethics and Human Philosophy, Medical University of Lublin, Lublin, Poland

This year, 2012, marks the 260th anniversary of the birth and the 180th anniversary of the death of Antonio Scarpa, an acclaimed anatomist and neurologist. He discovered the naso-palatine nerve (Scarpa’s nerve), the membranous labyrinth [1], endolymph (liquor Scarpae), and the ganglion of the vestibular nerve (Scarpa’s ganglion). What is more, his observations on neuroanatomy are still valid [1, 2].

**Fig. 1:**
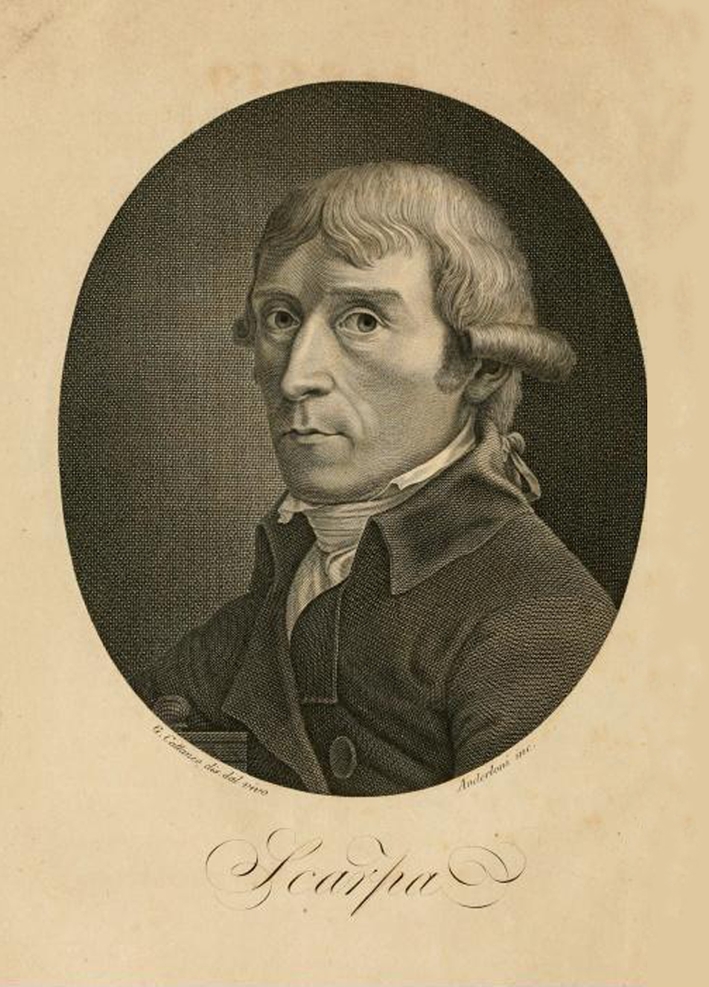
Antonio Scarpa (1752–1832). Reprinted from: Scarpa A (1801) Saggio di osservazioni e d’esperienze sulle principali malattie degli occhi. Presso Baldassare Comino, Pavia: frontpispiece

Antonio Scarpa (Fig. 1) was born on May 9, 1752 in Lorenzaga di Motta di Livenza, in the north-eastern region of Italy. At the age of 18, he graduated with honours in medicine at the University of Padua. Thanks to the support of his teacher and mentor, Giovanni Battista Morgagni, the young scholar became professor and head of the department of anatomy and surgery at the University of Modena just two years later, in 1772 [2–4]. After his appointment, he travelled to the Netherlands, France, and England. In 1783, Emperor Joseph II of Austria appointed him Professor of Anatomy at the University of Pavia [3]. In 1805, after Napoleon had been crowned King of Italy, he visited Pavia and inquired about the renowned anatomist Scarpa. Upon learning that Scarpa had been dismissed from the University because of his refusal to swear allegiance to the new king, Napoleon ordered to restore him in his position.

Through his achievements in neuroanatomy, Scarpa became an honorary member of the Royal Society of London in 1791 and of the Royal Swedish Academy of Sciences in 1821. He was an excellent lecturer; thanks to his fluent Latin he earned the nickname “magister eloquentiae maximae”. Notes from Scarpa’s lectures formed a complete textbook of surgery, clearly separating theoretical and practical knowledge. Scarpa never married, although he is said to have fathered several illegitimate children. At the end of his life he suffered from a urinary stone, which caused inflammation and subsequently led to his death on October 31, 1832. At his post-mortem, conducted by his former assistant Carlo Beolchin, Scarpa’s head, thumb and index finger were cut off, his urinary tract was removed and Scarpa’s assistants produced anatomical specimens of these body parts. Scarpa’s head is still kept as a memento of this eminent scientist in the Museo per la storia dell’Università di Pavia [2, 4].

Despite extraordinary achievements in the field of medicine, Scarpa’s marble statue was defaced soon after his death. This was probably caused by his arrogance, since he liked to emphasize his superiority. He ruthlessly challenged potential rivals and spread rumours about their alleged criminal activities. For positions at the university, he favoured friends and illegitimate sons.

Nevertheless, Scarpa’s flaws should not overshadow his achievements in the field of anatomy, especially neuroanatomy. His most important discoveries are the membranous labyrinth, the vestibular nerve ganglion (Scarpa’s ganglion) and the naso-palatine nerve [2–4]. Scarpa’s ganglion consists of bipolar cells, receiving impulses from the membranous labyrinth, utricle and saccule and continuing as vestibulocochlear nerve.

The naso-palatine nerve (Scarpa’s nerve) is the longest branch of the posterior parasympathetic pterygopalatine ganglion (Meckel’s ganglion), connected to the maxillary nerve (V2). It innervates the mucous membranes of the nasal cavity and partially those of the paranasal sinuses and also the autonomic glands and corpora cavernosa in this area. Scarpa was one of the first to draw attention to an affection of the inner coat of the arteries, now called atherosclerosis [5]. He identified the anatomical area on the thigh formed by the sartorius muscle, the adductor longus muscle and the inguinal ligament, currently known as Scarpa’s triangle. As a surgeon, Scarpa devoted much attention to aneurysms and hernia operations (he also described the sliding hernia). He was also interested in pediatric surgery and described the congenital clubfoot [3, 4].

Scarpa’s treatise on the anatomy of the middle ear was published as early as 1772, two years after graduation [6]. Most of his anatomical work, however, was published from Pavia, including the work on the anatomy of hearing and olfaction [7] and on the anatomy and diseases of the osteoarticular system [8]. His work on anatomy and diseases of the eyes from 1801 [9] assured him the title of “Father of Italian Ophthalmology”, also since it was the first publication on ophthalmology in the Italian language. For example, Scarpa described cataract treatment by depression rather than by extraction and a method of making artificial pupils. He also recommended a surgical treatment for dropsy of the eyeball [3, 9].

In 1794, Scarpa [10] published a collection of tables in which he presented the result of over 20 years of research on the nervous system. This work reveals Scarpa’s extraordinary artistic talent, since the vast majority of the figures were made by Scarpa himself. They are rich in detail and precisely outline the actual anatomical relations. Faustino Anderloni, an illustrator trained by Scarpa, also contributed. In this work, Scarpa [10] presented in a 1:1 ratio the vagus, glossopharyngeal and hypoglossal nerves. These nerves had never before been graphically presented with such precision and accuracy. Also for the first time in history he presented the nerves of the heart and showed that the terminal ramifications of the cardiac nerves are directly connected to the muscle fibres of the heart [3, 5, 10]. Other anatomists (e.g., Samuel Thomas von Sömmering) had already shown that the blood vessels of the heart are accompanied by nerves, but Scarpa should be credited with the discovery that cardiac muscle itself is supplied with nerves [3]. Sömmering had also noted that the nerves of the heart were smaller than those accompanying arteries of voluntary muscles [3], but Scarpa showed that within muscle tissue of either kind the nerves were of the same structure [3, 10]. Scarpa’s achievements in neuroanatomy deserve to be recalled, even 180 years after the death of this eminent scientist.
